# Prognostic value of elastic lamina staining in patients with stage III colon cancer

**DOI:** 10.1186/s12957-022-02865-y

**Published:** 2022-12-12

**Authors:** Feifei Bi, Xiaoyan Li, Yong Zhang, Zekun Wang, Qian Dong, Jingdong Zhang, Deyu Sun

**Affiliations:** 1 Medical Oncology Department of Gastrointestinal Cancer, Cancer Hospital of China Medical University, Liaoning Cancer Hospital & Institute, Shenyang, China; 2 Department of Pathology, Cancer Hospital of China Medical University, Liaoning Cancer Hospital & Institute, Shenyang, China; 3 Department of Medical Imaging, Cancer Hospital of China Medical University, Liaoning Cancer Hospital & Institute, Shenyang, China; 4 Department of Radiation Oncology Gastrointestinal and Urinary and Musculoskeletal Cancer, Cancer Hospital of China Medical University, Liaoning Cancer Hospital & Institute, Shenyang, China

**Keywords:** Colon cancer, Elastic lamina staining, Elastic lamina invasion, Disease-free survival, Recurrence risk

## Abstract

**Objective:**

The objectives of this study were to analyze the difference between the preoperative radiological and postoperative pathological stages of colorectal cancer (CRC) and explore the feasibility of elastic lamina invasion (ELI) as a prognostic marker for patients with stage III colon cancer.

**Methods:**

A total of 105 consecutive patients underwent radical surgery (R0 resection) for stage III colon cancer at the Cancer Hospital of China Medical University between January 2015 and December 2017. Clinicopathological features, including radiological stage and elastic lamina staining, were analyzed for prognostic significance in stage III colon cancer.

**Results:**

A total of 105 patients with stage III colon cancer who met the criteria and had complete data available were included. The median follow-up period of survivors was 41 months. During the follow-up period, 33 (31.4%) patients experienced recurrence after radical resection, and the 3-year disease-free survival (DFS) rate was 64.8%. The consistency between preoperative radiological and postoperative pathological staging was poor (κ = 0.232, *P* < 0.001). The accuracy of ≤ T2 stage diagnoses was 97.1% (102/105), that of T3 stage was 60.9% (64/105), that of T4a stage was 68.6% (72/105) and that of T4b stage was 91.4% (96/105). The DFS rate of T3 ELI (+) patients was significantly lower than that of both T3 ELI (−) patients (*P* = 0.000) and pT4a patients (*P* = 0.013). The DFS rate of T3 ELI (−) patients was significantly higher than that of pT4b patients (P=0.018). T3 ELI (+) (HR (Hazard ratio), 8.444 [95% CI, 1.736–41.067]; *P* = 0.008), T4b (HR, 57.727[95% CI, 5.547-600.754]; *P* = 0.001), N2 stage (HR, 10.629 [95% CI, 3.858–29.286]; *P* < 0.001), stage III (HR, 0.136 [95% CI, 0.31–0.589]; *P* = 0.008) and perineural invasion (PNI) (HR, 8.393 [95% CI, 2.094–33.637]; *P* = 0.003) were independent risk factors for postoperative recurrence of stage III colon cancer.

**Conclusions:**

The consistency between preoperative radiological and postoperative pathological staging was poor, especially for tumors located in the ascending colon and descending colon. Elastic lamina staining is expected to become a stratified indicator of recurrence risk for patients with stage III colon cancer and a guide for individualized adjuvant chemotherapy, thus improving patient prognosis.

## Background

Colorectal cancer (CRC) is one of the leading causes of cancer-related deaths worldwide. The CRC mortality rate is the third highest among both men and women [1]. With advancements in diagnostic and treatment methods, the incidence of CRC and the mortality of CRC patients have slowly decreased. However, CRC patient prognoses remain poor due to various high-risk factors. The TNM system was determined by the American Joint Committee on Cancer (AJCC) and the Union for International Cancer Control (UICC) and is an important tool for evaluating the prognosis of CRC patients and guiding treatment decisions [2]. SEER database data indicate that patients with stage III CRC have different prognoses depending on their stage type. The 5-year survival rates of colon cancer patients with stage IIIA, IIIB and IIIC disease are 62–71%, 38–55%, and 20–39%, respectively. Colectomy with en bloc removal of the regional lymph nodes is typically recommended for patients with resectable nonmetastatic CRC. However, neoadjuvant therapy is not routinely offered to patients with colon cancer because of the higher mortality rate resulting from chemotherapy toxicity and complications [3]. According to the NCCN guidelines, preoperative therapy is recommended only for T4b patients, resulting in significant downstaging, which is critical for colon cancer patients at high risk for recurrence [4]. Therefore, it is necessary to screen patients at high risk for relapse and provide individualized therapy during the perioperative period. The recurrence-free survival (RFS) rate (53%) of high-risk patients (T3 tumor beyond the border of the muscularis propria ≥ 5 mm or T4 stage) diagnosed preoperatively using computed tomography (CT) was significantly lower than that of low-risk patients (T1/T2) and moderate-risk patients (T3 tumor beyond the border of the muscularis propria < 5 mm) (87%) [5, 6]. In the FOxTROT trial, researchers evaluated patients stratified for recurrence risk at the preoperative radiological evaluation and indicated that high-risk patients have significantly improved prognoses and survival rates after receiving neoadjuvant therapy [3]. Therefore, preoperative radiology for risk-based stratification of colon cancer patients can help identify patients with a high risk for recurrence. This concept represents an individualized therapy approach for high-risk patients and further improves colon cancer patient prognoses.

Postoperative clinicopathological staging is the gold standard for diagnosis. However, some studies have shown substantial differences between the preoperative radiological stage and the postoperative pathological stage [4, 7, 8]. The accuracy of T4 stage determination is only 47%, and 50% of patients are staged too high (T4 at the preoperative radiological evaluation and pT3 at the postoperative pathological evaluation) [3]. On the one hand, radiologists consider minimal pericardial fat stranding due to benign desmoplastic reaction as tumor invasion to decrease the risk for understaging [7]. On the other hand, invasive tumors approaching the peritoneum often cause inflammation, mesothelial hyperplasia and fibrosis, which can obscure the anatomical markers of normal peritoneum [9]. Sometimes, hematoxylin-eosin (HE) staining cannot accurately show whether the peritoneum has been invaded. Thus, pathological staging of colon cancer is challenging.

Therefore, another traditional method, elastic lamina staining, has attracted widespread attention. Elastic lamina invasion (ELI) is a hallmark of pleural invasion and is considered in the clinical diagnosis and treatment of lung cancer [10]. The peritoneum and pleura have similar anatomical features. Therefore, elastic lamina staining can be used as an indicator to evaluate CRC prognosis. In colon cancer, the subserosal elastic lamina is located above the peritoneum [11]. Whether tumors have invaded tissues near the serosa can be evaluated by elastic lamina staining; such invasion is associated with poor prognoses in colon cancer patients [11–15]. Therefore, it has been suggested that CRC patients with ELI-positive (ELI+) pT3 tumors and those with pT4a tumors receive the same treatment [16]. For tumors in which the peritoneal invasion status cannot be accurately determined by HE staining, elastic lamina staining can be used to effectively determine the infiltration depth; thus, elastic lamina staining is expected to become a new anatomical method replacing traditional serosal invasion diagnostic approaches.

The objectives of this study were to analyze the difference between the preoperative radiological and postoperative pathological stages of CRC and explore the feasibility of ELI as a prognostic marker for patients with stage III colon cancer.

## Methods

### Study design and participants

A total of 105 consecutive patients who underwent radical surgery (R0 resection) for stage III colon cancer at the Cancer Hospital of China Medical University between January 2015 and December 2017 were included. Patients who underwent palliative surgery (R1, R2 resection), had other primary cancers in addition to colon cancer, and received neoadjuvant chemotherapy or radiation therapy were excluded from the study. The evaluated patient characteristics were age, sex, tumor location, preoperative radiological assessment (T stage, extramural vascular invasion (EMVI)), intestinal obstruction, postoperative pathological assessment (depth of penetration (T), number of lymph nodes positive (N) stage, grade of the cancer, pathological classification, lymphovascular invasion, perineural invasion (PNI)), and follow-up information. Disease-free survival (DFS) was defined as the time between the date of R0 resection and the date of recurrence or death due to disease progression. The enhanced computed tomography (CT) films and pathological sections were reviewed by a specialist gastrointestinal radiologist and pathologist blinded to the clinical follow-up information and the original assessment. Research ethics board approval (N0.20170223) was obtained from the Cancer Hospital of China Medical University, Shenyang, China.

### Pathological analysis

**Fig. 1:**
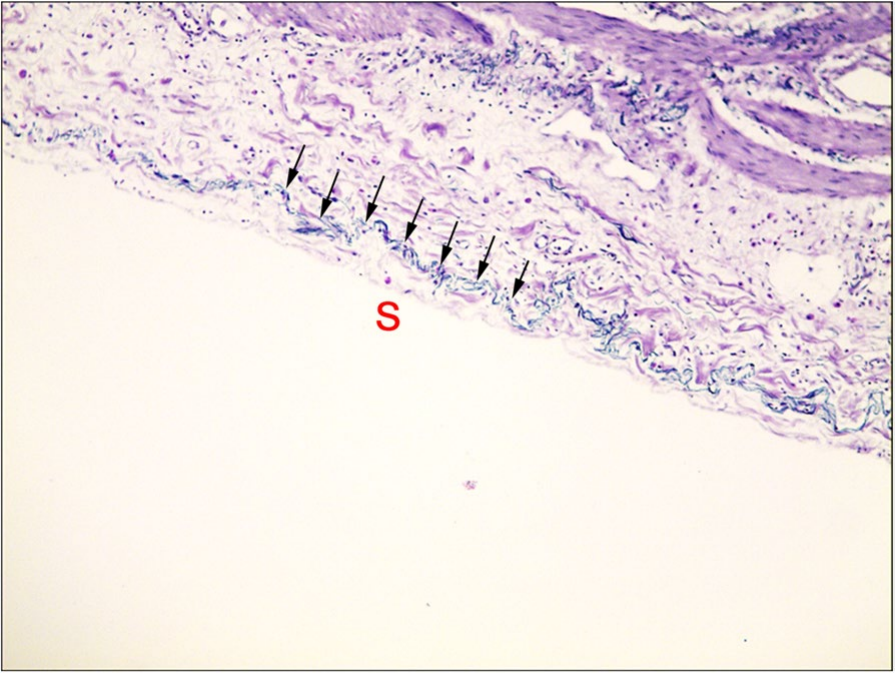
Elastic lamina staining of normal colon tissue (Fig. 1: × 100). Elastic lamina in the subserosal area; S: serosal surface. The black arrow indicates the elastic lamina

**Fig. 2:**
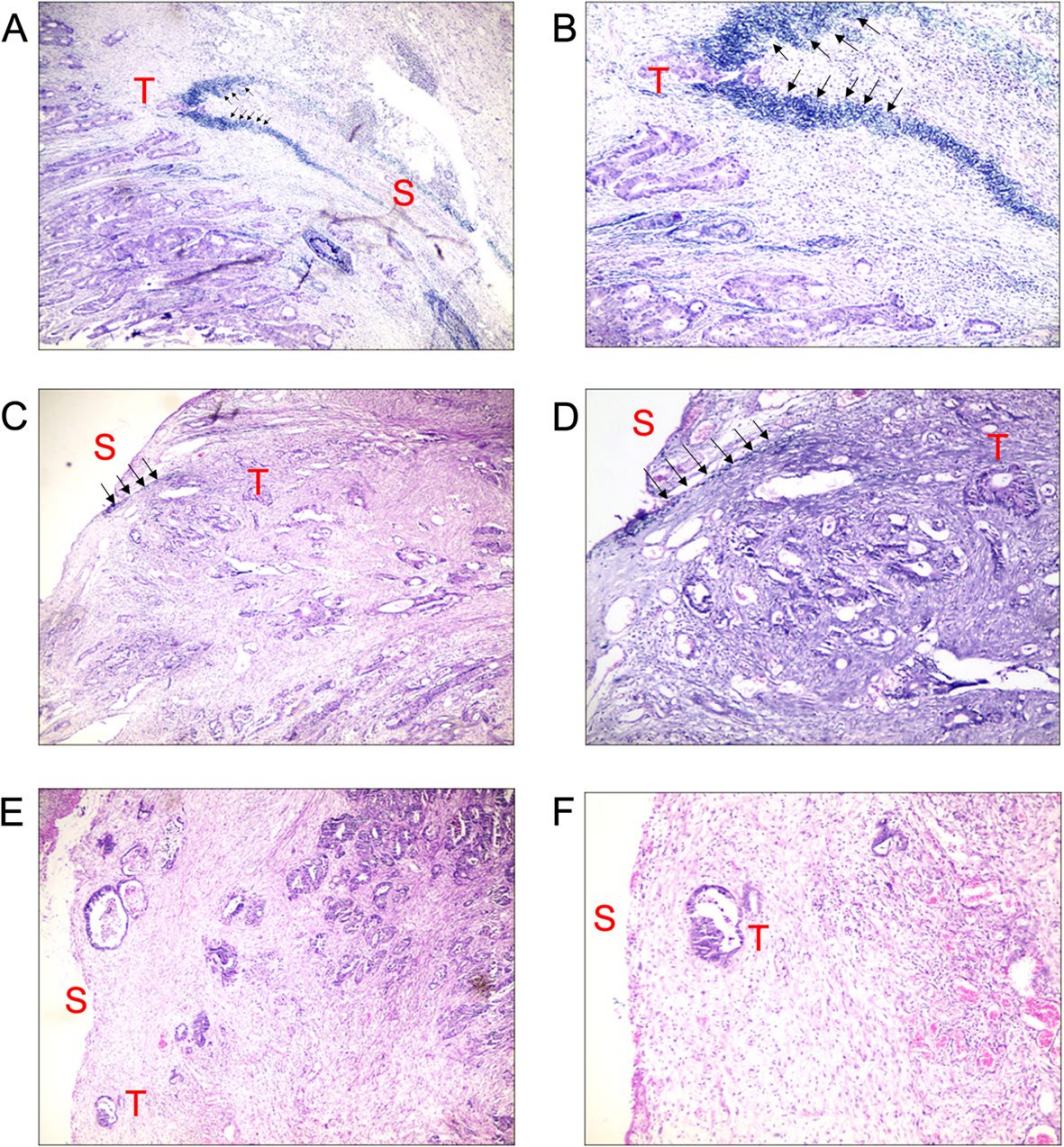
Elastic lamina staining of patient tissue sections. **A**, **B** The elastic lamina showed a “ring pattern”, and tumor cells invaded the elastic lamina, evaluated as pT3 ELI (+). S: serosal; T: tumor. The black arrow indicates the elastic lamina; (**A**: × 40, **B**: × 100). **C**, **D** The tumor approached the serosa but did not penetrate the elastic lamina, evaluated as pT3 ELI (−); S: serosal; T: tumor. The black arrow indicates the elastic lamina; (**C**: × 40, **D**: × 100). **E**, **F** The tumor approached the serosa but showed no staining for the elastic lamina, evaluated as pT3 EI (−); S: serosal; T: tumor. The black arrow indicates the elastic lamina; (E: ×40, F: × 100)

We chose one block from each case with the deepest tumor invasion level closest to the peritoneum according to the original HE staining assessment. We sliced at least one section from the block from each case and then stained the sections using the Van Gieson method. pT3 tumors were categorized as ELI positive (ELI+), ELI negative (ELI−), or EL not identified (EL−) based on the relationship between the tumor and elastic lamina (EL) (Figs. 1 and 2). ELI was defined as positive in the 4 following conditions [9]: (1) the tumor clearly invaded the elastic lamina; (2) the tumor invaded one side of the broken elastic lamina; (3) cellular mucin of mucinous tumors penetrated the elastic lamina; and (4) it invaded the outermost elastic lamina. Tumors with cells close to the serosa but not penetrating the elastic lamina were defined as ELI negative (ELI−).

### Radiological assessment

The preoperative radiological assessment standard is based on the 8th TNM of the AJCC. EMVI assessment was used to divide patients into negative and positive groups based on previous quantitative evaluations of EMVI in colon cancer [17, 18]. The tumors were graded as follows: grade 0 when the tumor invaded the colon wall but not the adjacent blood vessels; grade 1 when the tumor invaded a small area of the colon wall; grade 2 when the tumor invaded near a blood vessel but no tumor signal was found in the blood vessel; grade 3 when there was a tumor signal in the vascular cavity and the vascular cavity was dilated; and grade 4 when there were tumor signals in the vascular cavity and irregular new blood vessels arising from large blood vessels. Grades 0~2 were considered EMVI negative, and grades 3~4 were considered EMVI positive.

### Statistical analysis

SPSS 25.0 was used for statistical analyses. ANOVA and chi-square tests were used to analyze the differences between groups. The differences between preoperative radiological and postoperative pathology T stages were compared by kappa consistency analysis. Kaplan–Meier analysis and the log-rank test were used to evaluate the differences in DFS rates. Cox regression analyses were used to analyze the associations between independent factors and prognosis. A *P* value less than or equal to 0.05 was considered statistically significant.

## Results

### Patient characteristics

A total of 105 patients with stage III colon cancer were included in this study. The patient characteristics are shown in Table 1. The median age of the patients was 60 years (range, 34–85 years); 54 of the patients were male (51.4%). The tumors were located in the cecum in 3 (2.9%) patients, the ascending colon in 25 (23.8%), the hepatic flexure in 9 (8.6%), the transverse colon in 7 (6.7%), the splenic flexure in 5 (4.8%), the descending colon in 9 (8.6%) and the sigmoid colon in 47 (44.8%). Sixty-two (59%) patients had left-sided colon tumors, and 43 (41%) had right-sided colon tumors. Radiology revealed that 18 (17.1%) tumors were EMVI-positive, and 4 (3.8%) patients had intestinal obstruction before surgery. There were 44 (41.9%) patients with high-grade tumors and 61 (58.1%) with low-grade tumors. Thirteen (12.4%) patients had lymphovascular-invading tumors, and 5 (4.8%) tumors demonstrated PNI. The median follow-up period of survivors was 41 months (range, 4–69 months). A total of 101 (96.2%) patients received adjuvant chemotherapy after primary resection, and 33 (31.4%) patients experienced recurrence after radical resection.

**Table 1:** Clinicopathologic features of stage III colon cancer patients

Feature	Patients (*N* = 105)
Age (years) (median)	60
Sex	
Male	54 (51.4%)
Female	51 (48.6%)
Tumor location	
Cecum	3 (2.9%)
Ascending	25 (23.8%)
Hepatic flexure	9 (8.6%)
Transverse	7 (6.7%)
Splenic flexure	5 (4.8%)
Descending	9 (8.6%)
Sigmoid	47 (44.8%)
Location	
Left side of colon	62 (59%)
Right side of colon	43 (41%)
EMVI	
Positive	18 (17.1%)
Negative	87 (82.9%)
Intestinal obstruction	
Present	4 (3.8%)
Absent	101 (96.2%)
Pathology type	
Adenocarcinoma	74 (70.5%)
Mucinous adenocarcinoma	31 (29.5%)
Tumor differentiation	
Well	15 (14.3%)
Moderate	70 (66.7%)
Poorly to undifferentiated	20 (19%)
Tumor grade	
High	44 (41.9%)
Low	61 (58.1%)
Lymphovascular invasion	
Present	13 (12.4%)
Absent	92 (87.6%)
PNI	
Present	5 (4.8%)
Absent	100 (95.2%)
Adjuvant chemotherapy	
Yes	101 (96.2%)
No	4 (3.8%)
Recurrence	
Yes	33 (31.4%)
No	72 (68.6%)

### Comparison between preoperative radiological and postoperative pathological T stages

A total of 105 patients with colon cancer were reviewed by a specialist gastrointestinal radiologist. The evaluation results were as follows: 1 (1%) in cT1 stage, 0 (0%) in cT2 stage, 13 (12.4%) in cT3 < 5 mm, 55 (52.4%) in cT3 ≥ 5 mm, 26 (24.8%) in cT4a stage, and 10 (9.5%) in cT4b stage. According to postoperative pathological diagnosis, the accuracy of ≤ T2 stage diagnoses was 97.1% (102/105), that of T3 stage was 60.9% (64/105), T4a stage was 68.6% (72/105), and T4b stage was 91.4% (96/105) (Table 2). The accuracy of CT staging of tumors located in the cecum, ascending colon, hepatic flexure, transverse colon, splenic flexure, descending colon and sigmoid colon was 66.7%, 52%, 66.7%, 71.4%, 60%, 55.5%, and 59.6%, respectively (Table 3). According to the Kappa consistency analysis, the consistency between preoperative radiological and postoperative pathological staging was poor (κ = 0.232, *P* < 0.001).

**Table 2:** Consistency analysis of T stages between preoperative radiological stage and postoperative pathological stage

T stage	≤T2	T3	T4a	T4b
Sensibility	25% (1/4)	70.7% (46/65)	34.7% (8/23)	53.8% (7/13)
Specificity	100% (101/101)	45% (18/40)	78% (18/82)	96.7% (89/92)
Positive predictive value	100% (1/1)	67.6% (46/68)	30.7% (8/26)	70% (7/10)
Negative predictive value	97.1% (101/104)	48.6% (18/37)	81% (64/79)	93.6% (89/95)
Accuracy	97.1% (102/105)	60.9% (64/105)	68.6% (72/105)	91.4% (96/105)

**Table 3:** Consistency analysis of tumor location between preoperative radiological stage and postoperative pathological stage

Tumor location	Cecum	Ascending	Hepatic flexure	Transverse	Splenic flexure	Descending	Sigmoid
Insufficient assessment (cT<pT)	0 (0%)	4 (16%)	1 (11.1%)	2 (28.5%)	2 (40%)	1 (11.1%)	11 (23.4%)
Excessive assessment (cT>pT)	1 (33.3%)	8 (32%)	2 (22.2%)	0 (0%)	0 (0%)	3 (33.3%)	8 (17%)
Coincident assessment (cT=pT)	2 (66.7%)	13 (52%)	6 (66.7%)	5 (71.4%)	3 (60%)	5 (55.5%)	28 (59.6%)
Total	3	25	9	7	5	9	47

**Fig. 3:**
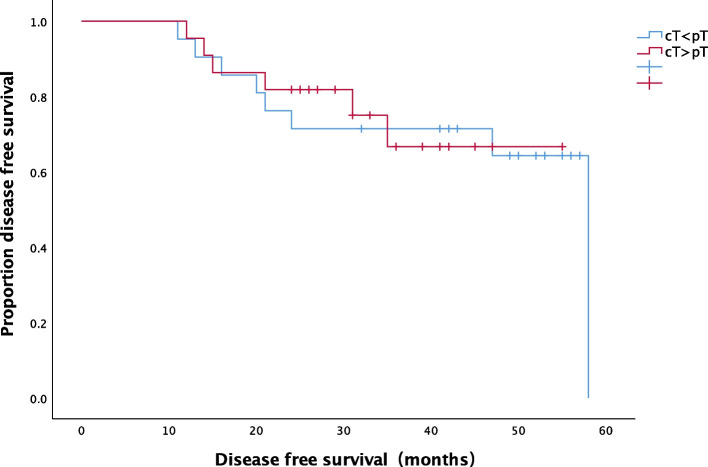
DFS based on the differences between preoperative radiology and postoperative pathology T stages

Patients were divided into the following groups based on the comparison between preoperative and postoperative staging: cT<pT (insufficient assessment; 20% of the patients [21]), cT>pT (excessive assessment; 21% of the patients [22]), and cT=pT (coincident assessment; 59% of the patients [62]). The clinicopathological features of these three groups are shown in Table 4. Analysis of variance and chi-square tests revealed no significant differences in age, sex, tumor location, N stage, EVMI, and intestinal obstruction among the groups (*p* > 0.05) (Table 4). The differences between preoperative radiology and postoperative pathology T stages had no significant effect on the DFS rate (*P* = 0.908) (Fig. 3).

**Table 4:** Clinicopathologic features of differences between preoperative radiology and postoperative pathology

Feature	cT< pT	cT> pT	cT=pT	*P*
Age (years) Median (range)	60 (37–81)	62 (38–85)	60 (34–81)	0.658
Sex				
Male	11 (52.4%)	11 (50%)	32 (51.6%)	0.987
Female	10 (47.6%)	11 (50%)	30 (48.4%)	
Tumor location				
Cecum	0 (0%)	1 (4.5%)	2 (3.2%)	0.722
Ascending	4 (19%)	8 (36.4%)	13 (21%)	
Hepatic flexure	1 (4.8%)	2 (9.1%)	6 (9.7%)	
Transverse	2 (9.5%)	0 (0%)	5 (8.1%)	
Splenic flexure	2 (9.5%)	0 (0%)	3 (4.8%)	
Descending	1 (4.8%)	3 (13.6%)	5 (8.1%)	
Sigmoid	11 (52.4%)	8 (36.4%)	28 (45.2%)	
Location				
Right side of colon	7 (33.3%)	11 (50%)	25 (40.3%)	0.533
Left side of colon	14 (66.7%)	11 (50%)	37 (59.7%)	
N stage				
N1	17 (81%)	14 (63.6%)	54 (87.1%)	0.055
N2	4 (19%)	8 (36.4%)	8 (12.9%)	
EVMI				
Positive	6 (28.6%)	4 (18.2%)	8 (12.9%)	0.255
Negative	15 (71.4%)	18 (81.8%)	54 (87.1%)	
Intestinal obstruction				
Present	0 (0%)	1 (4.5%)	3 (4.8%)	0.594
Absent	21 (100%)	21 (95.5%)	59 (95.2%)

### Application of elastic lamina staining for determining infiltration depth

In this study, 65 patients had T3 stage tumors, and 55 of these tumors were stained for elastic lamina analysis. There were 26 (47.3%) patients with T3 EL (−) tumors, 17 (30.9%) patients with T3 ELI (−) tumors and 12 (21.8%) patients with T3 ELI (+) tumors. Analysis of variance and chi-square tests revealed significant differences in the N stage among these groups (Table 5). There was significant difference in the N stage between T3 EL (−) and T3 ELI (+) patients (*P* = 0.024). There was significant difference in the N stage between T3 ELI (−) and T3 ELI (+) patients (*P* = 0.006). There was no significant difference in the N stage between T3 EL (−) and T3 ELI (−) patients (*P* = 0.342).

**Table 5:** Clinicopathological features of T3 EL (−), T3 ELI (+), and T3 ELI (−)

Features	T3 EL (−)	T3 ELI (+)	T3 ELI (−)	*P*
Age (years) Median (range)	63 (35–85)	62 (43–78)	62 (50–76)	0.615
Sex				
Male	14 (53.8%)	5 (41.7%)	8 (47.1%)	0.768
Female	12 (46.2%)	7 (58.3%)	9 (52.9%)	
Tumor location				
Cecum	1 (3.8%)	1 (8.3%)	0 (0%)	0.13
Ascending	4 (15.4%)	3 (25%)	6 (35.3%)	
Hepatic flexure	1 (3.8%)	0 (0%)	4 (23.5%)	
Transverse	3 (11.5%)	0 (0%)	0 (0%)	
Splenic flexure	1 (3.8%)	0 (0%)	2 (11.8%)	
Descending	2 (7.7%)	2 (16.7%)	1 (5.9%)	
Sigmoid	14 (53.8%)	6 (50%)	4 (23.5%)	
Location				
Right-sided colon	8 (30.8%)	4 (33.3%)	10 (58.8%)	0.161
Left-sided colon	18 (69.2%)	8 (66.7%)	7 (41.2%)	
N stage				
N1	22 (84.6%)	6 (50%)	16 (94.1%)	0.01
N2	4 (15.4%)	6 (50%)	1 (5.9%)	
Pathology type				
Adenocarcinoma	18 (69.2%)	7 (58.3%)	15 (88.2%)	0.174
Mucinous adenocarcinoma	8 (30.8%)	5 (41.7%)	2 (11.8%)	
Tumor differentiation				
Well	6 (23.1%)	1 (8.3%)	2 (11.8%)	0.656
Moderate	18 (69.2%)	9 (75%)	12 (70.6%)	
Poorly to undifferentiated	2 (7.7%)	2 (16.7%)	3 (17.6%)	
Tumor grade				
High	2 (7.7%)	2 (16.7%)	3 (17.6%)	0.568
Low	24 (92.3%)	10 (83.3%)	14 (82.4%)	
Lymphovascular invasion				
Present	0 (0%)	2 (16.7%)	2 (11.8%)	0.128
Absent	26 (100%)	10 (83.3%)	15 (88.2%)	
PNI				
Present	0 (0%)	1 (8.3%)	1 (5.9%)	0.371
Absent	26 (100%)	11 (91.7%)	16 (94.1%)	
EMVI				
Positive	2 (7.7%)	3 (25%)	1 (5.9%)	0.205
Negative	24 (92.3%)	9 (75%)	16 (94.1%)	

**Fig. 4:**
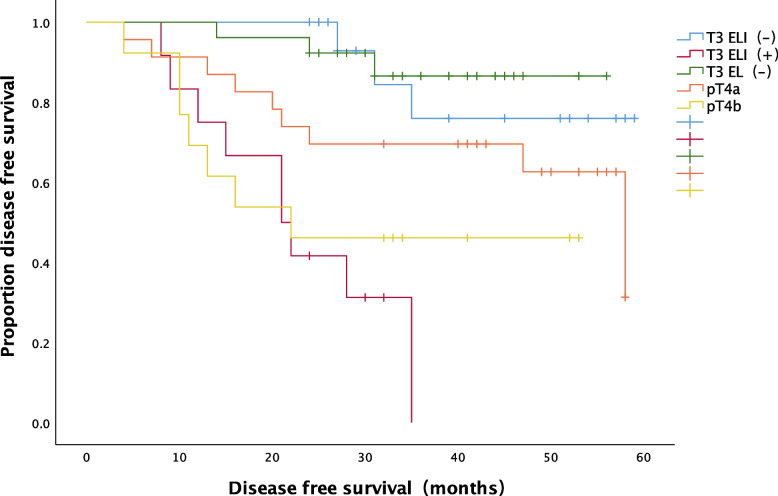
Association between DFS and tumor infiltration depth

Elastic lamina staining was performed on a total of 55 tumors from colon cancer patients with T3 stage tumors. Twenty-six (47.3%) of these patients had T3 EL (−) tumors, 17 (30.9%) had T3 ELI (−) tumors and 12 (21.8%) had T3 ELI (+) tumors. There were 23 patients with pT4a tumors and 13 with pT4b tumors. The DFS rate of T3 ELI (+) patients was significantly lower than that of T3ELI (−) patients (*P* = 0.000). The DFS rate of T3 ELI (+) patients was significantly lower than that of pT4a patients (*P* = 0.013). The DFS of T3 ELI (−) patients was significantly higher than that of pT4b patients (*P* = 0.018). There was no significant difference in the DFS rate between pT4b and T3 ELI (+) patients (*P* = 0.462). There was no significant difference in the DFS rate between pT4a and T3 ELI (−) patients (*P* = 0.158). There was no significant difference in the DFS rate between pT4a and pT4b patients (*P* = 0.165) (Fig. 4). We performed Cox regression analyses on all 105 patients with stage III colon cancer (Table 6). In the univariate analysis, T3 ELI (+) (*P* = 0.002), T4b (*P* = 0.016), N2 stage (*P* < 0.001), clinical stage (*P* = 0.017), nonadenocarcinoma pathological type (*P* = 0.033), lymphovascular invasion (*P* = 0.004) and PNI (*p* = 0.003) were associated with disease recurrence. The multivariate analysis suggested that T3 ELI (+) ([hazard ratio, HR], 8.444 [95% CI, 1.736–41.067]; *P* = 0.008), T4b (HR, 57.727[95% CI, 5.547–600.754]; *P* = 0.001), N2 stage (HR, 10.629 [95% CI, 3.858-29.286]; *P* < 0.001), stage IIIC (HR, 0.136 [95% CI, 0.31–0.589]; *P* = 0.008), and PNI (HR, 8.393 [95% CI, 2.094–33.637]; *P* = 0.003) were independent risk factors for postoperative recurrence of stage III colon cancer.

**Table 6:** Univariate and multivariate analyses of clinicopathological features and DFS

Feature	Univariate		Multivariate	
	HR (95% CI)	*P*	HR (95% CI)	*P*
Age (years)				
≤ 60	1	0.439	–	–
> 60	1.313 (0.659–2.614)			
Sex				
Male	1	0.96	–	–
Female	1.017 (0.514–2.016)			
ELI detection				
T3ELI (−)	1	0.001	1	< 0.001
T3ELI (+)	8.284 (2.177–31.521)		8.444 (1.736–41.067)	0.008
T3EL (−)	0.789 (0.157–3.963)		0.891 (0.158–5.026)	0.891
T4a	2.505 (0.677–9.263)		2.734 (0.625–11.958)	0.182
T4b	5.383 (1.367–21.194)		57.727 (5.547–600.754)	0.001
Tumor location				
Right-sided	1	0.802	-	-
Left-sided	1.094 (0.543–2.202)			
N stage				
N1	1	<0.001	1	< 0.001
N2	5.605 (2.786–11.273)		10.629 (3.858–29.286)	
Clinical stage				
IIIA	1	0.017	–	–
IIIB	0.862 (0.115–6.443)		1	0.008
IIIC	2.463 (0.320–18.960)		0.136 (0.31–0.589)	
Pathology type				
Adenocarcinoma	1	0.033	1	0.426
Mucinous adenocarcinoma	2.112 (1.063–4.197)		1.421 (0.599–3.372)	
Tumor grade				
Low	1	0.172	–	–
High	1.750 (0.784–3.903)			
Lymphovascular invasion				
Absent	1	0.004	1	0.25
Present	3.248 (1.457–7.241)		1.827 (0.654–5.104)	
PNI				
Absent	1	0.003	1	0.003
Present	4.980 (1.173–14.312)		8.393 (2.094–33.637)	
Adjuvant chemotherapy				
Yes	1	0.488	–	–
No	1.660 (0.397–6.950)			

## Discussion

Features such as tumor infiltration depth, lymph node metastasis, and EMVI are significantly associated with poor prognosis among colon cancer patients. The overall survival rate of patients with T4 stage colon cancer without lymph node metastasis is significantly lower than that of patients with T1–2 stage with lymph node metastasis [19, 20, 21]. Patients with T3/T4 stage are more likely to experience local regional recurrence or distant metastasis [22]. Some studies have revealed that patients at high risk for recurrence require more aggressive treatments, including more extensive lymph node dissection and hyperthermia and intraperitoneal chemotherapy (HIPEC) [23]. Therefore, identification of patients at high risk for recurrence who will benefit from individualized treatment is essential for improving colon cancer patient prognosis and survival rates. However, in this study, the consistency between preoperative radiological and postoperative pathological staging was poor (κ = 0.232, *P* < 0.001). The accuracy of T3 stage diagnosis was 60.9% (64/105), while that of T4a staging was 68.6% (72/105), which were consistent with the results of other studies [3, 4, 7]. Moreover, the accuracy of staging of the ascending colon and the descending colon tumors was poorer than that of tumors located in other colon sites. Furthermore, an excessive assessment phenomenon was present in ascending colon and descending colon tumors. Therefore, preoperative radiological staging may be insufficient for correctly identifying patients at high risk for recurrence [22]. On the one hand, due to the special anatomical structure of the colon, tumor staging by radiology is challenging, in particular in the peritoneal mesothelial organs (ascending and descending colon). The posterior walls of the ascending and descending colons are not covered with peritoneum, and the T stages at these sites were only T3 or T4b. On the other hand, when the colon wall is surrounded by a large amount of fat, it is difficult to distinguish fibroinflammatory reaction, connective tissue proliferation and tumor invasion in radiology [7], substantially impacting staging by preoperative radiology. Some studies have shown that MRI (magnetic resonance imaging) has higher diagnostic consistency than CT scan in locally advanced colon cancer, which can better identify serosal invasion [22, 24, 25]. Therefore, it is possible to combine CT and MRI preoperatively to improve the consistent rate of staging diagnosis and better identify patients with high recurrence risk.

Pathologically, it is difficult to determine whether tumor cells have invaded the peritoneum [26]. In previous studies, the diagnostic approaches for evaluating colon cancer with serosal invasion were varied and consistent [27]. On the one hand, mesenteric serosa surrounded by fat is difficult to identify. Furthermore, suspected sites of peritoneal invasion usually include peritoneal fracture and retroperitoneal structures, which makes it difficult for the pathologist to obtain samples and accurately determine the location of the peritoneum [12]. On the other hand, invasive tumors that approach the peritoneum often cause inflammation [28], including recruitment of inflammatory cells, such as CD68 and CD204 macrophages, to the vicinity of the serosa [12]. It is difficult to diagnose peritoneal invasion by H&E staining. Evaluating elastic lamina invasion performs better in terms of determining overall survival rates than evaluating tumor infiltration depth [12]; thus, elastic lamina staining is expected to become a new anatomical hallmark replacing traditional serosa invasion diagnostics. In this study, we not only found a higher risk for recurrence among T3 ELI (+) patients than for T3ELI (−) patients (*P* = 0.000) but also a higher risk for recurrence among T3ELI (+) patients than for pT4a patients (*P* = 0.013). T3ELI (+) (HR, 8.444 [95% CI, 1.736–41.067]; *P* = 0.008) was an independent risk factor for postoperative recurrence of stage III colon cancer. This may be related to the microenvironment of tumor metastases [29, 30]. Some studies have shown that the sites where tumors invade the elastic lamina usually show a greater extent of fibrosis and tumor budding [12, 13, 31]. Therefore, when the invasive tumor approaches the serosa, the tissue fibrosis and tumor buds increase rapidly. This process can form a tumor microenvironment that promotes the development of the tumor [13].

Previous studies involving elastic lamina staining have revealed a high rate of inability to detect the elastic lamina, partially casting doubt on whether this approach can be used to effectively evaluate the prognosis of colon cancer patients [9]. In this study, the elastic lamina was not detected in as high as 47.3% of the samples, and this effect was related to the N stage of the tumors. It has been shown that the number of negative nodes is an important independent prognostic factor for patients with stage IIIB and IIIC colon cancer [32]. Therefore, lymph node metastasis was essential for the prognosis of patients with stage III colon cancer. Our studies founded that there was no significant difference in the N stage between T3 EL (−) and T3 ELI (−) patients (*P* = 0.342). It might be suggested that the prognosis of T3 EL (−) was common with T3 ELI (−). Some studies have suggested similar views, they founded that there was no significant difference in the DFS (*p* = 0.6318) or OS (*p* = 0.8413) between the ELI (−) and the EL (−) [33]. With regard to the higher inability of elastic lamina detection. On the one hand, the tumor infiltrates the tissues near the serosa and produces fibroinflammatory and mesothelial reactions. This leads to the rupture and morphological distortion of the elastic lamina, which subsequently cannot be well identified. Moreover, the elastic lamina form a “ring pattern” (moving from the lower endothelial layer to the tumor, and then back to the lower endothelial layer), which further increases the difficulty of their identification [9]. On the other hand, the elastic lamina does completely cover the colon wall and its thickness varies with the anatomical location [34]. Some studies have shown that the identification rate of elastic lamina in the right side of the colon is lower than that in the left side of the colon [9, 35], and the identification rate in the rectum is lower than that in the colon [13]. Therefore, in other to avoid a high rate of elastic lamina identification failure, the pathological sampling and staining methods and diagnostic criteria need to be further developed. In clinical practice, serosal destruction by surgical resection of tumor tissue and lymph nodes is inevitable. Therefore, we advocate marking the suspected serosal invasion location on the specimen resected by surgery, allowing pathologists to carefully sample the suspected serosal invasion location. The elastic lamina may not be identified as it does not completely cover the colon wall. Therefore, the number of blocks and slices taken affects the rate of elastic lamina identification [13]. However, despite several studies, there are still no definitive guidelines regarding the number of blocks and slices needed. Furthermore, excessive sample collection and staining will cause unnecessary waste. Therefore, it may be beneficial to choose patients with T3 stage tumors with peritoneal invasion for elastic lamina staining [15]. The elastic lamina has been found to move from the nontumor area of the peritoneum to the muscularis propria when the tumor invades normal tissues [11]. It is possible to track the elastic lamina from the nontumor area to the tumor area. Therefore, rate of elastic lamina identification failure can be reduced more effectively. However, the materials used for elastic lamina staining and the criteria for diagnosis are inconsistent. We expect future prospective studies to standardize the pathological approach to diagnostics using elastic lamina staining.

For patients with stage III colon cancer and according to the NCCN guidelines, 12 cycles of FOLFOX4/mFOLFOX6 or 8 cycles of XELOX postoperative adjuvant chemotherapy have been recommended if they can tolerate intensive treatment. Patients who cannot tolerate oxaliplatin should consider capecitabine or 5-FU monotherapy. However, the disease-free survival (DFS) and overall survival (OS) rates of patients at high risk for relapse after adjuvant chemotherapy are obviously different. The results of the MOSAIC 10-year study showed that compared with that of N2 stage patients, the 5-year DFS rate of N1 stage patients increased by 16% (72.3% vs. 55.4%), and the 10-year OS rate increased by 12% (71.4% vs. 59.5%) [36]. Therefore, it is challenging to stratify recurrence risk and optimize treatment for patients with stage III colon cancer. In the 2017 IDEA study, patients with stage III colon cancer were divided into low-risk (T1, T2, T3N1) and high-risk (T4 or N2) groups. Studies have shown that the 3-month DFS rate in the high-risk group is lower the 6-month DFS rate (73.6% vs. 76.0%), while there is no difference between the 3- and 6-month DFS rates in the low-risk group. (83.1% vs. 83.3%). Additionally, different chemotherapy strategies have different prognoses. The DFS rate after 3 months of FOLFOX treatment is lower than that after 6 months of treatment, while CAPOX for 6 months in the low-risk group does not provide a treatment benefit compared with 3 months of therapy [37]. Therefore, NCCN guidelines recommend FOLFOX for 6 months or CAPOX for 3–6 months for high-risk patients with stage III colon cancer, and CAPOX for 3 months and FOLFOX for 3–6 months for low-risk patients with stage III colon cancer. However, the patient clinical features significantly related to prognosis among high-risk individuals include RAS/BRAF mutation and MMR status in addition to clinical stage. In recent years, diagnosis with circulating tumor DNA (ctDNA) has improved due to its noninvasiveness, convenience, safety, and comprehensive nature. ctDNA has application value in the diagnosis of early colorectal cancer, monitoring postoperative early recurrence, and monitoring treatment response and therapeutic resistance in patients with metastatic disease. It remains unclear whether patients with stage III colon cancer have a high risk for recurrence with postoperative adjuvant chemotherapy. Some studies have shown that ctDNA analyses can be used to assess patient risk considering postoperative adjuvant chemotherapy and guide individualized follow-up strategies [38]. The results of the phase III IDEA-France clinical trial revealed that 13.65% of patients had ctDNA-positive status before postoperative chemotherapy. In the low-risk group, the prognosis of ctDNA-positive patients after 3 months of treatment was poorer than that of ctDNA-negative patients. Therefore, further prospective clinical trials are required to define the clinicopathological features of high-risk groups.

Elastic lamina staining can not only effectively diagnose the depth of tumor infiltration but can also indicate recurrence risk in patients with stage III colon cancer after radical resection. Elastic lamina staining results can further stratify the recurrence risk of patients with stage III colon cancer, and prospective clinical trials are required to determine optimal adjuvant treatment strategies. It is expected that elastic lamina staining will be of diagnostic and treatment value for patients with colon cancer.

## Data Availability

All data generated or analyzed during this study are included in this published article.

## Abbreviations

CRC: Colorectal cancer
AJCC: American Joint Committee on Cancer
UICC: Union for International Cancer Control
RFS: Recurrence-free survival
CT: Computed tomography
HE: Hematoxylin-eosin
ELI: Elastic lamina invasion
ELI+: ELI positive
ELI−: ELI negative
EL: Elastic lamina
EL−: Elastic lamina not identified
EMVI: Extramural vascular invasion
PNI: Perineural invasion
DFS: Disease-free survival
ANOVA: Analysis of variance
HR: Hazard ratio
HIPEC: Hyperthermia and intraperitoneal chemotherapy
MRI: Magnetic resonance imaging
MMR: Mismatch repair
ctDNA: Circulating tumor DNA
